# Supplementary material for: Pyruvate Dehydrogenase Kinase 1 inhibition mediated oxidative phosphorylation enhancement in cartilage promotes osteoarthritis progression
Source: BMC Musculoskelet Disord. 2023 Jul 20;24:597. doi: 10.1186/s12891-023-06585-6 (PMC10357736; doi:10.1186/s12891-023-06585-6)
Supplement: Supplementary file 1 — Additional file 1. [file 12891_2023_6585_MOESM1_ESM.zip › BP.Gsea.1653623667859/GOBP_CELL_FATE_DETERMINATION.html]

Details for gene set GOBP\_CELL\_FATE\_DETERMINATION[GSEA]

|  || Dataset | 55457\_collapsed\_to\_symbols.cls.cls #OA\_versus\_normal.cls.cls #OA\_versus\_normal\_repos |
| Phenotype | cls.cls#OA\_versus\_normal\_repos |
| Upregulated in class | normal |
| GeneSet | GOBP\_CELL\_FATE\_DETERMINATION |
| Enrichment Score (ES) | -0.5721817 |
| Normalized Enrichment Score (NES) | -2.0914307 |
| Nominal p-value | 0.0 |
| FDR q-value | 0.01176061 |
| FWER p-Value | 0.073 |
Table: GSEA Results Summary

  

Fig 1: Enrichment plot: GOBP\_CELL\_FATE\_DETERMINATION      
 Profile of the Running ES Score & Positions of GeneSet Members on the Rank Ordered List

  

| SYMBOL | TITLE | RANK IN GENE LIST | RANK METRIC SCORE | RUNNING ES | CORE ENRICHMENT || 1 | PAX6 | paired box 6 [Source:HGNC Symbol;Acc:HGNC:8620] | 304 | 0.746 | 0.0251 | No |
| 2 | FEZF2 | FEZ family zinc finger 2 [Source:HGNC Symbol;Acc:HGNC:13506] | 2278 | 0.329 | -0.1067 | No |
| 3 | ISL1 | ISL LIM homeobox 1 [Source:HGNC Symbol;Acc:HGNC:6132] | 2373 | 0.318 | -0.0933 | No |
| 4 | PRRX1 | paired related homeobox 1 [Source:HGNC Symbol;Acc:HGNC:9142] | 3657 | 0.189 | -0.1806 | No |
| 5 | MEF2C | myocyte enhancer factor 2C [Source:HGNC Symbol;Acc:HGNC:6996] | 4218 | 0.135 | -0.2153 | No |
| 6 | PROX1 | prospero homeobox 1 [Source:HGNC Symbol;Acc:HGNC:9459] | 4299 | 0.128 | -0.2131 | No |
| 7 | ATOH1 | atonal bHLH transcription factor 1 [Source:HGNC Symbol;Acc:HGNC:797] | 4428 | 0.119 | -0.2153 | No |
| 8 | GATA6 | GATA binding protein 6 [Source:HGNC Symbol;Acc:HGNC:4174] | 5537 | 0.030 | -0.2995 | No |
| 9 | PAX2 | paired box 2 [Source:HGNC Symbol;Acc:HGNC:8616] | 6564 | -0.047 | -0.3762 | No |
| 10 | LBX1 | ladybird homeobox 1 [Source:HGNC Symbol;Acc:HGNC:16960] | 6570 | -0.047 | -0.3735 | No |
| 11 | WNT7A | Wnt family member 7A [Source:HGNC Symbol;Acc:HGNC:12786] | 7166 | -0.093 | -0.4137 | No |
| 12 | ASCL1 | achaete-scute family bHLH transcription factor 1 [Source:HGNC Symbol;Acc:HGNC:738] | 7375 | -0.108 | -0.4228 | No |
| 13 | BMP4 | bone morphogenetic protein 4 [Source:HGNC Symbol;Acc:HGNC:1071] | 7816 | -0.142 | -0.4477 | No |
| 14 | MYOD1 | myogenic differentiation 1 [Source:HGNC Symbol;Acc:HGNC:7611] | 7839 | -0.143 | -0.4400 | No |
| 15 | POU6F2 | POU class 6 homeobox 2 [Source:HGNC Symbol;Acc:HGNC:21694] | 8308 | -0.179 | -0.4647 | No |
| 16 | FOXG1 | forkhead box G1 [Source:HGNC Symbol;Acc:HGNC:3811] | 8356 | -0.182 | -0.4565 | No |
| 17 | PTCH1 | patched 1 [Source:HGNC Symbol;Acc:HGNC:9585] | 8837 | -0.220 | -0.4794 | No |
| 18 | NKX2-2 | NK2 homeobox 2 [Source:HGNC Symbol;Acc:HGNC:7835] | 8977 | -0.230 | -0.4751 | No |
| 19 | PTCH2 | patched 2 [Source:HGNC Symbol;Acc:HGNC:9586] | 8994 | -0.231 | -0.4613 | No |
| 20 | TRIM15 | tripartite motif containing 15 [Source:HGNC Symbol;Acc:HGNC:16284] | 10156 | -0.337 | -0.5295 | No |
| 21 | WNT1 | Wnt family member 1 [Source:HGNC Symbol;Acc:HGNC:12774] | 10706 | -0.394 | -0.5464 | Yes |
| 22 | NOTCH2 | notch receptor 2 [Source:HGNC Symbol;Acc:HGNC:7882] | 10720 | -0.395 | -0.5216 | Yes |
| 23 | SOX17 | SRY-box transcription factor 17 [Source:HGNC Symbol;Acc:HGNC:18122] | 10843 | -0.410 | -0.5043 | Yes |
| 24 | JAG1 | jagged canonical Notch ligand 1 [Source:HGNC Symbol;Acc:HGNC:6188] | 10977 | -0.427 | -0.4867 | Yes |
| 25 | CYP26B1 | cytochrome P450 family 26 subfamily B member 1 [Source:HGNC Symbol;Acc:HGNC:20581] | 11022 | -0.433 | -0.4618 | Yes |
| 26 | GATA2 | GATA binding protein 2 [Source:HGNC Symbol;Acc:HGNC:4171] | 11058 | -0.439 | -0.4358 | Yes |
| 27 | IFRD1 | interferon related developmental regulator 1 [Source:HGNC Symbol;Acc:HGNC:5456] | 11170 | -0.453 | -0.4148 | Yes |
| 28 | HOXA2 | homeobox A2 [Source:HGNC Symbol;Acc:HGNC:5103] | 11183 | -0.455 | -0.3860 | Yes |
| 29 | NOTCH4 | notch receptor 4 [Source:HGNC Symbol;Acc:HGNC:7884] | 11441 | -0.495 | -0.3737 | Yes |
| 30 | TBX2 | T-box transcription factor 2 [Source:HGNC Symbol;Acc:HGNC:11597] | 11728 | -0.538 | -0.3607 | Yes |
| 31 | GATA3 | GATA binding protein 3 [Source:HGNC Symbol;Acc:HGNC:4172] | 12276 | -0.670 | -0.3595 | Yes |
| 32 | HES1 | hes family bHLH transcription factor 1 [Source:HGNC Symbol;Acc:HGNC:5192] | 12676 | -0.849 | -0.3350 | Yes |
| 33 | CDC42 | cell division cycle 42 [Source:HGNC Symbol;Acc:HGNC:1736] | 12681 | -0.852 | -0.2796 | Yes |
| 34 | KLF4 | Kruppel like factor 4 [Source:HGNC Symbol;Acc:HGNC:6348] | 12848 | -1.058 | -0.2234 | Yes |
| 35 | EBF2 | EBF transcription factor 2 [Source:HGNC Symbol;Acc:HGNC:19090] | 12859 | -1.088 | -0.1530 | Yes |
| 36 | CTNNB1 | catenin beta 1 [Source:HGNC Symbol;Acc:HGNC:2514] | 12866 | -1.119 | -0.0803 | Yes |
| 37 | MCL1 | "MCL1 apoptosis regulator, BCL2 family member [Source:HGNC Symbol;Acc:HGNC:6943]" | 12892 | -1.267 | 0.0006 | Yes |
Table: GSEA details [plain text format]

  

Fig 2: GOBP\_CELL\_FATE\_DETERMINATION      
 Blue-Pink O' Gram in the Space of the Analyzed GeneSet

  

Fig 3: GOBP\_CELL\_FATE\_DETERMINATION: Random ES distribution      
 Gene set null distribution of ES for **GOBP\_CELL\_FATE\_DETERMINATION**
